# AI-Assisted Decision-Making in Long-Term Care: Qualitative Study on Prerequisites for Responsible Innovation

**DOI:** 10.2196/55962

**Published:** 2024-07-25

**Authors:** Dirk R M Lukkien, Nathalie E Stolwijk, Sima Ipakchian Askari, Bob M Hofstede, Henk Herman Nap, Wouter P C Boon, Alexander Peine, Ellen H M Moors, Mirella M N Minkman

**Affiliations:** 1 Vilans Centre of Expertise of Long Term Care Utrecht Netherlands; 2 Copernicus Institute of Sustainable Development Utrecht University Utrecht Netherlands; 3 Human Technology Interaction Eindhoven University of Technology Eindhoven Netherlands; 4 Faculty of Humanities Open University of The Netherlands Heerlen Netherlands; 5 TIAS School for Business and Society Tilburg University Tilburg Netherlands

**Keywords:** decision support systems, ethics, long-term care, responsible innovation, stakeholder perspectives

## Abstract

**Background:**

Although the use of artificial intelligence (AI)–based technologies, such as AI-based decision support systems (AI-DSSs), can help sustain and improve the quality and efficiency of care, their deployment creates ethical and social challenges. In recent years, a growing prevalence of high-level guidelines and frameworks for responsible AI innovation has been observed. However, few studies have specified the responsible embedding of AI-based technologies, such as AI-DSSs, in specific contexts, such as the nursing process in long-term care (LTC) for older adults.

**Objective:**

Prerequisites for responsible AI-assisted decision-making in nursing practice were explored from the perspectives of nurses and other professional stakeholders in LTC.

**Methods:**

Semistructured interviews were conducted with 24 care professionals in Dutch LTC, including nurses, care coordinators, data specialists, and care centralists. A total of 2 imaginary scenarios about AI-DSSs were developed beforehand and used to enable participants articulate their expectations regarding the opportunities and risks of AI-assisted decision-making. In addition, 6 high-level principles for responsible AI were used as probing themes to evoke further consideration of the risks associated with using AI-DSSs in LTC. Furthermore, the participants were asked to brainstorm possible strategies and actions in the design, implementation, and use of AI-DSSs to address or mitigate these risks. A thematic analysis was performed to identify the opportunities and risks of AI-assisted decision-making in nursing practice and the associated prerequisites for responsible innovation in this area.

**Results:**

The stance of care professionals on the use of AI-DSSs is not a matter of purely positive or negative expectations but rather a nuanced interplay of positive and negative elements that lead to a weighed perception of the prerequisites for responsible AI-assisted decision-making. Both opportunities and risks were identified in relation to the early identification of care needs, guidance in devising care strategies, shared decision-making, and the workload of and work experience of caregivers. To optimally balance the opportunities and risks of AI-assisted decision-making, seven categories of prerequisites for responsible AI-assisted decision-making in nursing practice were identified: (1) regular deliberation on data collection; (2) a balanced proactive nature of AI-DSSs; (3) incremental advancements aligned with trust and experience; (4) customization for all user groups, including clients and caregivers; (5) measures to counteract bias and narrow perspectives; (6) human-centric learning loops; and (7) the routinization of using AI-DSSs.

**Conclusions:**

The opportunities of AI-assisted decision-making in nursing practice could turn into drawbacks depending on the specific shaping of the design and deployment of AI-DSSs. Therefore, we recommend considering the responsible use of AI-DSSs as a balancing act. Moreover, considering the interrelatedness of the identified prerequisites, we call for various actors, including developers and users of AI-DSSs, to cohesively address the different factors important to the responsible embedding of AI-DSSs in practice.

## Introduction

### Background

For the long-term care (LTC) of older adults, technologies based on artificial intelligence (AI) are increasingly being developed and deployed to support the nursing process, from the assessment and diagnosis of care needs to the planning, implementation, and evaluation of care strategies addressing these needs [1-8]. For instance, AI-based decision support systems (AI-DSSs) can support specific aspects of the nursing process, such as monitoring the behavior and vital signs of clients with the aim of identifying frailty, assessing dementia-related problems and suitable interventions, and triaging health deterioration before eventually transferring clients to an emergency department or institutional care setting [1,9-13]. Throughout the nursing process, nurses, care coordinators, and other care professionals need to navigate a complex web of diagnostic and therapeutic uncertainties, client preferences and values, and cost considerations [14,15]. Against the backdrop of a growing gap between the number of qualified caregivers and the number of people in need of care, AI-assisted decision-making by caregivers could help sustain and improve the quality and efficiency of care.

AI-based technologies can, for explicit or implicit objectives, infer from the input they receive how to generate outputs such as predictions, content, recommendations, or decisions that can influence physical or web-based environments [16-18]. AI-DSSs refer to information systems that acquire relevant data about care needs or processes; present relevant data to users, such as nurses; and possibly translate raw data into actionable information, such as alerts, risk assessments, or recommendations about care strategies [15,19-21]. AI-based technologies such as AI-DSSs combine *preprogrammed, rule-based* algorithms and *data-driven, self-learning* algorithms rooted in machine learning. While initially rule focused, AI-DSSs are increasingly incorporating machine learning. This enables them to extract patterns and new insights from data sets that may be challenging for humans to analyze and improve their performance (eg, recommendations) based on the new data [2,15,21-23]. Therefore, the anticipated progress in AI-DSSs suggests their growing role in *proactively* supporting nurses and other stakeholders in decision-making regarding person-centered care strategies by harnessing relevant data.

Notwithstanding the potential of AI-DSSs and other AI-based technologies to support caregivers and other stakeholders in LTC, their deployment creates ethical and social challenges. The long-term gathering of data on the health and well-being of individuals, along with the pivotal role of algorithms in interpreting these data to arrive at care-related decisions, raises concerns. These concerns encompass the potential erosion of the privacy, autonomy, and self-determination of individuals; depersonalization of the caregiver-client relationship; and discrimination, problematization, and stigmatization of old age [4,21,24-27]. Owing to the impact that the use of AI-based technologies may have on the lives of older adults and the work of caregivers and the potential resistance that might emerge during implementation, implications need to be assessed and addressed at an early stage of their development.

In recent years, a growing prevalence of guidelines and frameworks to provide guidance on responsible AI innovation for diverse stakeholders, such as researchers, legislators, technology developers, and technology users, has been observed. Studies that have compared responsible AI frameworks emphasize a general consensus around high-level principles, such as transparency, justice, fairness, and nonmaleficence [28-30]. However, the current guidelines are generally highly abstract and leave much room for the interpretation of how these principles can be practically applied and contextualized to specific technologies, such as AI-DSSs, and specific settings, such as LTC [30,31]. Although scholars recognize the importance of a more context-specific conceptualization of these principles, multiple literature reviews have shown that only a few studies specify practical approaches to responsible AI innovation for specific application domains, which is particularly true for AI applications in LTC [5,7,32,33].

### This Study

This study aimed to fill this knowledge gap by presenting the results of an interview study on prerequisites for responsible AI-assisted decision-making in nursing practice, with a specific focus on the LTC domain. In-depth interviews were conducted with Dutch nurses and professional stakeholders (ie, care coordinators, data specialists, and care centralists) with whom nurses closely collaborate. This holds particular relevance because these stakeholders have firsthand experience and practical insights into the nursing processes where AI-DSSs are anticipated to play an increasing role. Thereby, they can contribute significantly to understanding both the potential impact of AI-DSSs and the factors that need to be addressed for the responsible embedding of these technologies in practice. While various studies have offered conceptual expert analyses and synthesized relevant literature on factors important to the responsible embedding of AI-DSSs in health care (eg, the studies by Heyen and Salloch [22], Hindocha and Badea [34], and Skuban-Eiseler et al [35]), few have investigated (future) user perspectives on responsible AI-assisted decision-making [36]. This study first examined the perspectives of nurses and other professional stakeholders in LTC on the opportunities and risks of AI-assisted decision-making in nursing practice, thereby laying the groundwork for the second and main objective: exploring prerequisites for responsible innovation in this area. The results can lead to recommendations for responsibly embedding AI-DSSs into nursing practice.

## Methods

### Overview

Semistructured interviews were conducted to explore the perspectives of nurses and other professional stakeholders in LTC regarding, first, the opportunities and risks of AI-assisted decision-making in nursing practice and, second, associated prerequisites for responsible innovation in this area. This approach enabled the researchers to delve deeply into specific areas of interest while also maintaining an open-ended format that encourages participants to share their perspectives freely. This was crucial to comprehending the rationale behind perceived opportunities and risks and, consequently, thoroughly exploring associated prerequisites for responsible innovation. The interviews were conducted as part of the Healthy Ageing Eco-system for People with Dementia (HAAL) project, which is part of the European Active and Assisted Living program (Active and Assisted Living Europe, 2021; project AAL-2020-7-229-CP). In HAAL, an international consortium consisting of care organizations, research institutes, and commercial firms from the Netherlands, Italy, Taiwan, and Denmark collaborates on the co-design, development, testing, and commercialization of an AI-DSS intended to provide actionable information to formal caregivers of frail older adults, particularly those with dementia, with the aim of reducing the caregiver workload and increasing the quality of care. The consortium acknowledges that innovators must anticipate, reflect on, and respond to the ethical and social implications of increasingly advanced AI-DSSs at an early stage of innovation. Therefore, in parallel with the iterative co-design, development, and field testing of a low-complexity AI-DSS, the empirical research presented in this paper was conducted to explore the prerequisites for responsible innovation in AI-DSSs.

As envisioning the potential impacts of using AI-DSSs can be challenging, we first used scenarios and then used principle-based probing themes as starting points to explore stakeholder perspectives on the potential impact of using AI-DSSs and prime interview participants toward reflecting on both opportunities and risks. A total of 2 distinct imaginary scenarios were developed as inputs for the interviews, outlining different roles of AI within AI-DSSs. The aim of the scenarios was to make abstract concepts such as AI and AI-DSSs more concrete, enabling interview participants to articulate their expectations and considerations regarding the opportunities and risks of AI-assisted decision-making in nursing practice more effectively [37-40]. The AI-DSS in the first scenario incorporates only descriptive analytical functions that examine data to uncover insights into past events or trends. This scenario was inspired by the AI-DSS developed in the HAAL project. The second scenario takes a more speculative and ambitious turn and involves a more advanced AI-DSS with descriptive, predictive, and prescriptive functions. Predictive functions analyze data to forecast future outcomes, and prescriptive functions analyze data to recommend specific actions or strategies to help achieve specific outcomes [41,42]. Thus, in comparison to the first scenario, the second scenario adopts a more proactive approach in supporting decision-making regarding person-centered care strategies.

In addition to the scenarios, specific principle-based probing themes were used to evoke thorough consideration of the risks of using AI-DSSs in LTC, along with possible strategies and actions to address or mitigate these risks in the design, implementation, and use of AI-DSSs. These probing themes were based on the six principles for responsible AI, as proposed by the World Health Organization (WHO) guidance on *Ethics and Governance of Artificial Intelligence for Health* [43]: (1) protecting human autonomy; (2) promoting human well-being and safety and the public interest; (3) ensuring transparency, explainability, and intelligibility; (4) fostering responsibility and accountability; (5) ensuring inclusiveness and equity; and (6) promoting AI that is responsive and sustainable. This particular guidance was selected because it represented one of the latest guidelines issued by an authoritative body in the health care domain. Moreover, it was explicitly designed as a starting point for context-specific discussions involving diverse stakeholders [43].

### Participants

In total, 24 participants took part in this study. Recruitment took place through email inquiries to care organizations involved in the HAAL project and other LTC facilities in the Netherlands. The researchers aimed to achieve a varied composition of participants with different roles in the LTC for older adults and varying degrees of experience with technology, data, and AI. The inclusion of diverse professional perspectives offers insights into different facets of care where AI-DSSs might play an increasing role and contributes to a multifaceted understanding of prerequisites for responsible AI-assisted decision-making in nursing practice.

Participants were broadly categorized into 4 groups: nurses (13/24, 54%), care coordinators (6/24, 25%), data specialists (3/24, 12%), and care centralists (2/24, 8%). Nurses had various roles and education levels, ranging from executive district nurses to quality nurses with responsibilities in the care coordination of different clients. The group of care coordinators, including dementia case managers (2/6, 33%), geriatric care coordinators (2/6, 33%), and specialists in geriatric care (2/6, 33%), primarily coordinated and oversaw various aspects of care for frail older adults, including medical, social, and support services. Data specialists play a central role within their care organization in using data and developing tools, such as dashboards, to support decision-making by care teams. Finally, nursing care centralists are positioned within care centers in the Netherlands that respond to alarms (eg, from active and passive alarm instruments) and care-related questions, for instance, by calling in a caregiver on-site when needed.

Of the 24 participants, 16 (67%) held a formal role in advancing digitization within their care organizations. This might imply that these participants had already made or could relatively easily make explicit representations of the opportunities and risks of AI-assisted decision-making in LTC and prerequisites for responsible innovation in this area. More specifically, these participants consisted of 9 (69%) of the 13 participating nurses, 2 (33%) of the 6 participating care coordinators, all 3 (100%) data specialists, and both (2/2, 100%) care centralists. Furthermore, of the 24 participants, 18 (75%) were female, and 6 (25%) were male. The mean age of the participants was 41 (SD 12.8; range 21-61) years, and on average, the participants had 16 (SD 11.4; range 3-40) years of occupational experience in health care.

### Procedure and Materials

All interviews were conducted digitally via video calls, with screen sharing used to provide visual support for the interview questions. The interviews were conducted between May 2022 and February 2023, with a mean duration of 79 (range 58-119) minutes. Of the 24 interviews, 17 (71%) were conducted by pairs of researchers, and 7 (29%) were conducted by a single researcher. A multidisciplinary group of researchers (DRML, NES, SIA, HHN, WPCB, and AP) developed the interview protocol. Minor adaptations were made to the protocol after pilot testing with the first 2 participants. The interviews were conducted in Dutch.

The interview protocol (Multimedia Appendix 1) was structured as follows. In the first part of the interviews, a general introduction was given about the AI-DSS developed in the HAAL project. This concerns a dashboard that acquires, presents, and uses data generated by various digital care and well-being technologies that can be deployed in the homes of older adults. When used, these technologies collect data on the physical activity, eating and sleeping patterns, cognitive functioning, mood, social contact, and medication intake of older adults. All technologies were explained and shown to participants using a visual illustration, and questions were asked about the perceived relevance of and the familiarity of participants with the various technologies and data.

In the second part, participants were invited to reflect on the opportunities of AI-assisted decision-making in LTC. A description and visual illustration were provided, and questions related to the 2 developed imaginary scenarios were asked. The first scenario describes a dashboard with descriptive analytical functions only. The dashboard provides an overview of the data collected over time via a tailored selection of digital care and well-being technologies. In the dashboard, specific collected data are marked by a color (red, orange, or green) to signify varying levels of risk or urgency associated with them. Apart from the application of this coloring scheme, the data are not interpreted by algorithms. The primary goal of this dashboard is to make the data generated by various technologies available to caregivers in one place to prevent them from looking at separate overviews and apps.

The second scenario describes a more advanced dashboard with descriptive, predictive, and prescriptive functions. In this scenario, the data generated by the selected care technologies are not only integrated into one system and color marked to signify risk levels but also automatically processed into actionable insights by algorithms. Actionable insights could entail predictions of the risk for future emergency situations, such as a fall, and recommendations about possible follow-up actions, such as stimulating the physical activity of a client if the data indicate a relatively inactive period.

Both scenarios left room for the interview participants to indicate whether, and for which types of caregivers and other stakeholders in LTC, the respective dashboard might be relevant and why. After questions in this regard, participants were asked which of the 2 dashboards they would prefer and why. In addition, a short explanation was provided about the term *AI*, including everyday examples, after which the participants were asked what role they hope AI will play in the future of LTC.

In the third part, the participants were asked about the risks related to the use of AI-DSSs in LTC, as well as mitigation strategies. Participants were first invited to openly discuss any risks or concerns linked to both scenarios and consider whether they perceived any explicit differences in the risks associated with more advanced AI-DSSs compared to low-complexity AI-DSSs. Subsequently, targeted questions about risks were asked by using the 6 probing themes based on the responsible AI principles from the WHO [43]. After a brief explanation of each principle, participants were asked about their views on the respective principle in the context of AI-assisted decision-making in LTC. During discussions of potential risks, participants were encouraged to brainstorm possible strategies and actions to address or mitigate these risks in the design, use, and implementation of AI-DSSs. Finally, the participants were asked whether they had any other suggestions or topics that they wanted to discuss regarding the implications of using AI-DSSs in LTC.

### Ethical Considerations

Before the interviews, general information about the goal and procedure was provided, and the participants were asked to read and sign an informed consent form. The authors of this study followed the guidelines in the Declaration of Helsinki and the Dutch code of conduct for scientific integrity. Ethical approval for the interviews, not subject to the medical scientific research act involving human subjects, was granted by an independent board of the lead author's department (Vilans), including a privacy officer and legal expert [44]. The recorded interviews were transcribed verbatim using a professional transcription service. The transcripts were thereafter coded for confidentiality, and identifying information was removed.

### Analyses

A thematic analysis was independently performed by 4 researchers using the MAXQDA 2022 (VERBI GmbH) analysis software. One researcher (DRML) analyzed all 24 transcripts, and 3 researchers (NES, SIA, and BMH) analyzed 8 transcripts each. While distributing tasks, the goal was to give each researcher the broadest possible view of the data set. Therefore, NES and SIA, who were involved in conducting some of the interviews, analyzed transcripts of interviews in which they had not been involved themselves. The transcripts were analyzed through a stepwise construction of codes. On the basis of our research objective, three initial main codes were established: (1) opportunities for AI-assisted decision-making in nursing practice, which were represented by potential supportive roles of AI-DSSs in this context; (2) risks of AI-assisted decision-making, which provide indications of factors that need to be addressed for the responsible embedding of AI-DSSs in practice; and (3) associated prerequisites for responsible AI-assisted decision-making, which were represented by strategies to mitigate specific risks in the design, implementation, and use of AI-DSSs. Our in-depth analysis of the transcripts followed the 6 steps outlined by Braun and Clarke [45] and comprised a largely inductive thematic analysis to identify, analyze, and report repeated patterns across the interview transcripts [46]. The WHO principles provided a predefined theoretical framework that informed our thematic analysis, yet, apart from the 3 initial main codes, the development of codes and subcodes was largely inductive and reflective for the pertinent issues raised by the data. During the coding process and after initial coding, all 4 researchers engaged in 3 consultation sessions to exchange and cross-validate interpretations and coding decisions among themselves, thereby fostering intercoder reliability. Some of the results were presented through illustrative quotes, which were translated from Dutch to English and carefully selected to represent the arguments presented in the interviews and justify the various perspectives shown in the interviews. During the selection process, we considered whether the quotes could be understood without the context in which they were originally uttered.

## Results

### Overview

This section presents the perspectives of participants on prerequisites for responsible AI-assisted decision-making in nursing practice. First, we thoroughly discuss the anticipated opportunities and risks of AI-assisted decision-making in nursing practice, as these established a foundation for the participants in exploring prerequisites for responsible innovation in this area. Thereafter, we discuss the associated prerequisites for responsible AI-assisted decision-making in nursing practice that were inductively identified.

### Opportunities and Risks of AI-Assisted Decision-Making in Nursing Practice

#### Overview

On the basis of their substantial experience and domain knowledge of LTC, all participants were able to make explicit representations of potential supportive roles of AI-DSSs in the nursing process. Most participants also discussed a diverse array of risks of using AI-DSSs in nursing practice, even though multiple participants shared that they lacked experience in contemplating the risks and disadvantages of using AI-DSSs and AI more broadly. Comments about risks were frequently raised spontaneously when participants were prompted to reflect on the 2 imaginary scenarios outlining different types of AI-DSSs. However, in most cases, these comments were shared as a response to either open or targeted (principle-based) interview questions about risks. Through our thematic analysis, involving open coding, it became evident that the identified opportunities and risks of AI-assisted decision-making in nursing practice coexist as complementary yet contradictory elements within 4 (interrelated) thematic domains: the early identification of care needs, guidance in devising care strategies, shared decision-making, and the workload and work experience of caregivers. For each of the domains, we discuss the opportunities and risks in the subsequent subsections.

#### Early Identification of Care Needs

Most participants anticipated that AI-DSSs could support caregivers in the remote and early anticipation of care needs, thereby enabling them to proactively initiate appropriate interventions. As multiple participants discussed, various existing care technologies enable caregivers to monitor the health, well-being, and behavior of clients remotely. The data generated by such technologies can provide insights into the changing care needs of specific clients. Such data could not only be remotely accessed and evaluated by caregivers but might also be automatically processed through AI into actionable insights, such as signals and alarms for caregivers about increased risks. Given that an increasing amount of data is being collected through various care technologies, multiple participants explicitly expressed optimism that AI could enable and optimize the use of these increasing amounts of data, thereby enhancing the already implemented and more stand-alone forms of remote monitoring. Furthermore, some participants perceived that insights gained through continuous technology-based monitoring might contribute to more adequate and complete information about care needs because, for instance, clients may not always (be able to) share all relevant information, the observations of caregivers when visiting clients generally provide only a limited view of the entire situation, and caregivers might inconsistently report on the same situation. One of the nurses shared the following:

If you think there is a specific care need but you are not sure what is actually happening in the client’s room or house, we now often still ask about the nurse’s gut feeling, which is often correct, of course, but now [with an AI-DSS] we can check with data what is really the case.

Participant 14

In this line, some participants suggested that AI-DSSs could assist caregivers in targeted risk assessments or attempts to gain insights into specific unexplained behavior of clients. Furthermore, some participants anticipated that AI, with its ability to discern subtle patterns from data, could swiftly uncover emerging trends or potentially overlooked areas of attention regarding the health, well-being, or behavior of a client.

Notwithstanding these opportunities, participants shared multiple concerns regarding the identification of care needs based on personal data. For instance, multiple participants stated that a false sense of security may be created when caregivers heavily rely on or excessively trust the outputs of AI-DSSs, assuming that these outputs encompass all relevant patterns regarding the health, well-being, and care needs of clients. In addition, some participants stated that numerous issues or concerns related to older adult data could be flagged as potentially problematic. As suggested, this might result in caregivers adopting care interventions, possibly under pressure from other stakeholders, such as the families of clients. However, these interventions may be perceived as unnecessary or even undesirable by stakeholders such as the clients themselves. Therefore, the use of AI-DSSs might lead to the over-problematization of old age and stigmatizing stereotypes, impacting both the quality of life of older adults and the workload of caregivers. One of the care coordinators stated the following:

The system may ignore the norms and values of a particular client...Sometimes things that may seem very problematic may actually not be that problematic to a client.

Participant 22

In addition, multiple participants commented that potential misuse of or unauthorized access to personal data could jeopardize the individual privacy of older adults; their ability to make their own decisions (ie, autonomy); and, consequently, their trust in their care network. Moreover, some participants suggested that the potential opacity of AI algorithms may complicate the understanding of both clients and caregivers of certain outcomes of AI-DSSs and care-related decisions made with the assistance of AI-DSSs. As suggested, this may diminish their trust and confidence in the collection and use of personal data. Furthermore, some participants commented that shifts toward data- and AI-assisted remote care might not be widely accepted. According to 2 participants, this raises questions regarding the extent to which enforcing these changes on clients or caregivers who are hesitant or unwilling to adopt these new approaches can be justified. One of the nurses expressed this as follows:

It [using AI-DSSs] becomes part of the foundation of your profession...It becomes an important part of determining your actions. But if someone does not want that, then you suddenly need your old-fashioned skills again, which requires a different way of caregiving that may no longer fit in with regular work processes or the zeitgeist...And then it could also be that the health insurer says: “We will no longer pay for that, because there is a better alternative.”

Participant 20

#### Guidance on Devising Care Strategies

Multiple participants anticipated that by pointing caregivers to possible care needs and providing inspiration or substantiation for suitable care strategies, AI-DSSs might increasingly guide or direct caregivers in decision-making regarding person-centered care strategies. As some participants commented, AI-DSSs might thereby act as a type of personal coach, mentor, or advisor with 3 apparent, related functions. First, multiple participants suggested that AI-DSSs may offer inspiration or evidence for tailored person-centered interventions aimed at improving the health and well-being of a client, thereby helping caregivers devise care strategies to address specific issues. Second, multiple participants envisioned that AI-DSSs could facilitate the substantiation and validation of the initial ideas of caregivers about care strategies by using objective data to reinforce why these strategies should be implemented or explored further. Third, some participants anticipated that AI-DSSs might increasingly support caregivers in evaluating whether certain person-centered interventions were, in retrospect, suitable and whether adjustments should be made. Thus, the AI-DSSs were anticipated to enable iterative data-informed deliberation on person-centered care strategies. Some participants suggested that AI-DSSs may be particularly useful for relatively inexperienced caregivers who may overlook certain matters or possible care strategies owing to a lack of experience or for temporary substitute workers who are less familiar with the behavior, daily rhythm, and personal needs or preferences of a client. Others stated that more experienced caregivers may also find value in such AI assistance because of their potentially deeply rooted approaches to understanding care needs and implementing care strategies that could be challenged by the output of AI-DSSs.

Despite these potential benefits, most participants also shared concerns that guidance by AI-DSSs in devising care strategies could lead to the overreliance of caregivers on these systems. Multiple participants stated that heavy reliance on AI-DSSs by caregivers may gradually diminish their capacity for independent decision-making and critical thinking about person-centered care. One of the nurses said the following:

What I find a bit scary when a system is many times more intelligent than you, is that it does not always necessarily make you smarter...The more you are facilitated with knowledge and interpretations and so on, the less you have to think for yourself.

Participant 21

In addition, some participants suggested that caregivers who rely heavily on AI-DSSs may insufficiently consider broader contextual factors or crucial nuances in the characteristics and needs of individual clients. One of the nurses explained this as follows:

For instance, a male client who is very autistic may often retreat to his room and feel good about that. I can imagine that the system would then say: “This client rarely leaves his room, there is a risk of loneliness.” Then you may think that is a good conclusion, while it is actually good for this man that he often withdraws himself. Otherwise, he would be seriously overstimulated.

Participant 11

As some participants expressed, heavy reliance on AI-DSSs might result in misguidance toward unsuitable care strategies and negative impacts on the overall quality of care owing to the reduced adaptability of caregivers and the care system as a whole to unforeseen circumstances or erroneous or suboptimal recommendations by AI-DSSs.

#### Shared Decision-Making

Several participants anticipated that AI-DSSs would support shared decision-making by older adults and their formal and informal caregivers. Multiple participants mentioned that AI-DSSs could support caregivers in conversations with clients and their care network, including informal and other formal caregivers, by helping clarify care needs, identify unaddressed care needs, and reveal and substantiate necessary adjustments in the care plan. Similar to the broader spectrum of data and technology, AI-DSSs are perceived as potential conversational tools, fostering a more collective approach to decision-making in nursing practice. A few participants also mentioned that the use of AI-DSSs could support the shared responsibility of different caregivers in providing good care. One of the nurses suggested the following:

A psychological side effect of sharing information amongst all care professionals is that care coordinators no longer feel solely responsible for difficult decisions such as scaling down care. It is increasingly becoming a shared responsibility. By sharing information and anchoring it in the process, there is much more support for difficult measures.

Participant 3

Simultaneously, it emerges from the comments of some participants that, instead of using data and AI-DSSs outcomes as input for shared decision-making, people might also intentionally or unintentionally use these outcomes against one another. As some participants expressed, in contexts where AI-DSSs collect, store, and use sensitive personal data, multiple interests could be intertwined and conflicting, such as the interest of a client in protecting their dignity and personal boundaries, the interest of professional caregivers in anticipating and understanding care needs, the interest of informal caregivers in monitoring (the quality of) formal care provided, and the interest of health insurers in exercising control over the care provided. Ultimately, conflicts of interest can result in mistrust.

#### Workload and Work Experience

Most participants suggested that the use of AI-DSSs might alleviate the cognitive load of caregivers and improve their work experience. Most participants envisioned that AI-DSSs could relieve caregivers of or even enable the processing of large amounts of pertinent data gathered in the care context. Some participants perceived it to be increasingly unrealistic to expect caregivers to invest time in tasks involving the analysis of substantial amounts of data, considering the high workload, the increasing amount of data gathered in the care context, and the lack of analytical skills to interpret these data. Accordingly, multiple participants suggested that AI-DSSs could relieve the workload of caregivers by automating routine tasks such as monitoring the daily rhythm or medication intake of the clients. In addition, some participants stated that by AI-DSSs taking on data-intensive and repetitive tasks, caregivers might experience a substantial decrease in mental strain and a more sustainable work environment. Furthermore, a few participants mentioned that a decrease in cognitive load resulting from the use of AI-DSSs might allow caregivers to dedicate more time and attention to empathetic aspects of caregiving and nuanced decision-making about person-centered care, rooted in thorough research into the specific care needs of clients.

In contrast, multiple participants suggested that the use of AI-DSSs might lead to an increased workload and deteriorate the work experience of caregivers. Some participants anticipated that caregivers using AI-DSSs might be unable to comprehend (some of) the outcomes of the systems or feel overwhelmed by the number of AI-generated insights, alarms, and recommendations for follow-up. Some participants also stated that caregivers might feel pressured to follow-up on the outcomes of the AI-DSSs. One of the nurses commented on this as follows:

I see the risk that if you as a care professional decide to ignore a system, like “I’ll let this one go” or “I don’t recognize this [problem] at all,” then it could become a difficult story...To what extent will you, as a care professional, still have the right to say: “I will not do this,” or “I see it differently?”

Participant 20

Furthermore, multiple participants mentioned that the heavy reliance of caregivers on AI-DSSs might diminish their active role and autonomy in investigating care needs and devising person-centered care strategies. Consequently, as some participants suggested, job satisfaction and the sense of professional fulfillment or purpose that caregivers could derive from person-centered and empathetic aspects of caregiving might be reduced.

### Prerequisites for Responsible AI-Assisted Decision-Making in Nursing Practice

#### Overview

Building upon the anticipated opportunities and risks of AI-assisted decision-making, participants discussed a broad array of factors that should be considered to responsibly embed AI-DSSs in nursing practice and optimally balance opportunities and risks. These factors can be roughly divided into 7 interrelated categories of prerequisites for responsible AI-assisted decision-making in nursing practice.

#### Regular Deliberation on Data Collection

Stakeholders in data practices, including clients, should regularly deliberate on the data required as inputs for AI-DSSs. Despite the potential of AI-DSSs to provide better insights as they acquire more (eg, more diverse or more long-term) data, most participants stressed that only essential data should be acquired to, for instance, limit privacy infringements, counteract the over-problematization of old age, and prevent the cognitive overload of caregivers. One of the nurses stressed this as follows:

What I personally find troubling is that we want to keep an eye on people all day long...I would rather like us to look more closely at specific points about which we say: we might want some extra attention on that. So, for example you might want to know more about—I’ll name it—the medication moment around ten o’clock. What happens around that moment that makes that the client may or may not do something with it? Or a fall incident, what happens before that makes the person fall every time?

Participant 21

Along this line, multiple participants advocated that the collection of data should always relate to specific objectives (ie, care needs or life goals) agreed upon by clients and caregivers. Some participants also proposed regular deliberation by stakeholders, including clients, on the necessity and implications of specific data collection, as care needs, the personal values of stakeholders, and technological possibilities change over time.

#### A Balanced Proactive Nature of AI-DSSs

AI-DSSs should have a balanced proactive nature, implying that they should proactively support the nursing process while avoiding decision automation. On the one hand, multiple participants stressed that AI-DSSs should ease data-intensive analytical tasks by processing data into actionable insights that encourage caregivers to implement certain care strategies or delve deeper into identified concerns. Some participants proposed that it is crucial to avoid overwhelming caregivers with excessive insights that, from a practical perspective (eg, owing to limited time and resources), cannot be acted upon or are not necessarily problematic.

On the other hand, there was a broad consensus among participants that human agency in decision-making should not be overshadowed and that ample space should be created for caregivers to devise person-centered care strategies by themselves. Multiple participants suggested that the need for users to think critically for themselves should be explicitly communicated to users during implementation. Some participants proposed that users could also be informed about this via the user interface of AI-DSSs. Furthermore, multiple participants noted that it could be meaningful if AI-DSSs point caregivers to specific areas of concern but that caregivers should largely retain and take the responsibility to develop person-centered approaches to address specific issues. One of the nurses stated the following:

If you see that a client has been less mobile the entire week, I think you should look at it like: “okay, what have we observed ourselves in recent weeks?”...And what actions you take in response, I think, always depends on the client...Let caregivers think for themselves about the interventions that are appropriate, because of course you do not always have to implement the same interventions in a certain situation.

Participant 7

#### Incremental Advancements Aligned With Trust and Experience

Advancements in AI-DSSs should involve incremental steps that align with users’ and other stakeholders’ evolving trust in, and experience with, these systems. Despite the perceived need for proactive AI-DSSs that can transform potentially unmanageable data sets into actionable insights, multiple participants stressed that their operation and use should provisionally not entail excessive complexity or opacity. Caregivers, clients, and other stakeholders should gradually build trust as AI-DSSs prove their value during use. Multiple participants envisioned that, as trust in and experience with AI-DSSs deepens, gradual advancements in these systems could be implemented. For instance, it may be useful to introduce more advanced predictive and prescriptive analytical functionalities provided that users can interact with the system without diminishing their autonomy and critical thinking abilities. In addition, some participants posited that before broader deployment, significant adjustments to algorithms and the underlying logic within AI-DSSs may first need to be extensively tested in a secure setting and evaluated by an independent body. One of the data specialists stated the following:

I think we need a quality mark to establish that trust and that we as sector must agree that if such a system does not have such a quality mark and it is still under development, we will not use it.

Participant 12

#### Customization for All User Groups

The design and implementation of AI-DSSs should involve customization for all user groups, including clients and caregivers, such that users’ interactions with AI-DSSs are tailored to their personal needs. Some participants stated that no one-size-fits-all approach exists for clients when deploying care technologies or collecting data related to their health, well-being, and care needs. Differences between clients regarding their views on what is important in life and what contributes to quality of care (eg, the best possible curative care, safety, freedom, and privacy) may need to translate into variations regarding the choice of care technologies to be deployed, the data collected as input for AI-assisted decision-making (see also prerequisite 1), and who can access the resulting insights. Similarly, multiple participants suggested that some degree of customization should also be applied to caregivers. The interaction of AI-DSSs with caregivers, for instance, the type of insights provided and the extent to which recommendations by the systems have already been concretized, and the training of caregivers to use AI-DSSs optimally may need to be tailored to the specific role, level of education, problem-solving capacities, and ability for critical reflection of the caregivers. One of the care coordinators stated the following:

I think it depends on the resolving power of the person viewing it...Non-medical caregivers level two can often care for people very kindly and can help with washing, dressing and providing pills. But you cannot expect that when a client is ill, they will understand what needs to be changed with those medicines. So then maybe there must be a signal [by an AI-DSS] saying “maybe you should discuss with the nurse or doctor what should be done with the medication.” But if you make that suggestion to a higher educated nurse, she will say “yes, duh, I know that. That is my profession.” It might quickly cause irritation if things go like that.

Participant 1

#### Measures to Counteract Bias and Narrow Perspectives

During the design and practical deployment of AI-DSSs, measures should be taken to counteract bias and narrow perspectives. In respect to the design of AI-DSSs, multiple participants suggested that transparency should be provided regarding the underlying functioning of AI-DSSs to ensure that caregivers can properly understand the generation of AI-based insights and assess the applicability and relevance of these insights in the context of an individual client. Simultaneously, some participants posited that, although a certain level of transparency is essential, it should not entirely hinder the advantages offered by advanced and potentially opaque AI analytics. Multiple participants suggested that transparency about AI-based outcomes could be fostered through explanations via the user interface of AI-DSSs about underlying trends in the data that led to a specific outcome or about the types of data and client characteristics considered to achieve certain outcomes. In addition, multiple participants proposed that the output of AI-DSSs be framed as advice rather than compelling information to prevent users from following AI-based outcomes without critical reflection. In addition, some participants suggested that, in cases where AI-DSSs provide caregivers with recommendations about interventions to address specific care needs, multiple possible strategies could be presented to prevent caregivers from fixating on a specific solution. Furthermore, some participants advocated incorporating contextual information about client characteristics, such as cultural and socioeconomic backgrounds, as well as the observations or interpretations of caregivers. Such information could provide a broader perspective on the relevance of specific AI-generated insights and might be crucial for caregivers to develop a nuanced understanding of the situation and care needs of a client. Moreover, multiple participants suggested that it might be relevant if AI-DSSs not only provide insight into areas of attention in the health and care of clients but also highlight positive trends that indicate, for instance, that a certain care intervention has been successful.

In respect to the practical deployment of AI-DSSs, most participants stressed that caregivers require training on the responsible use of these systems. For instance, multiple participants proposed training to critically evaluate the relevance of AI-generated insights and resist a potential tendency to accept supposedly “evidence-based” outputs from AI-DSSs as the truth. In addition, some participants stressed that training should counteract the possibility that caregivers overconcentrate on specific facets of health and well-being or particular care interventions to which AI-DSSs have guided their attention. One of the nurses stated the following:

I think it is important to indicate very clearly in the training, for example, that options are presented for what you can do, but that you are supposed to think for yourself about what fits. Are you going to adjust the action slightly, are you going to take a completely different action, or aren’t you going to anything at all?

Participant 17

#### Human-Centric Learning Loops

AI-assisted decision-making should involve human-centric learning loops, meaning that caregivers should be involved in both the design of AI-DSSs and their implementation and use in practice. One suggested aspect of such involvement is that caregivers could assist designers in determining and iteratively improving the underlying logics of AI-DSSs during both the initial design and practical use of these systems. Multiple participants advocated that caregivers with domain-specific knowledge and an affinity to technology assist designers, who may lack such contextual knowledge, in drawing up and testing assumptions regarding the conversion of specific data into meaningful insights to support nursing practice. In addition, a few participants suggested that caregivers could be involved in labeling or annotating data in the training data sets for AI-DSSs. Furthermore, some participants proposed that caregivers could reinforce the learning process of AI by assisting designers in ensuring that adaptive AI-DSSs adequately refine their outputs based on new data and user feedback. Similarly, multiple participants mentioned that caregivers who actually use AI-DSSs in practice should have the option to review AI-generated outcomes and provide feedback that reinforces their learning capabilities. For instance, some participants suggested enabling caregivers to set the specific threshold values from which a certain alarm should be generated for specific clients, indicate how they followed up on specific AI-generated insights and why, and manually enter relevant matters overlooked by the system. A nurse stated the following:

It may be good to have the possibility to also add information as a professional, important data that may affect the client and care...If someone does absolutely not want physiotherapy, but that is recommended by the system every time, then you want to be able to indicate somewhere that this is no longer an option, so that the system can take that into account, and look for a second best option.

Participant 6

Another suggested aspect of human-centric learning loops is that caregivers can support each other in the use of AI-based insights in practice. Several participants commented that caregivers who are progressive with and at the forefront of using AI-DSSs could be assigned the responsibility of facilitating the use of AI-DSSs by other caregivers who may lack experience, be hesitant to use AI-DSSs, or not know how to handle certain outcomes. Similarly, some participants suggested that, in the context of AI-assisted decision-making, it might be relevant or necessary to involve interdisciplinary care professionals who act as intermediaries between care and technology. As suggested, these professionals could assist less data-savvy caregivers in interpreting data and AI-based outputs to formulate care strategies.

#### Routinization of Using AI-DSSs

Finally, the use of AI-DSSs should become routine, promoting a commitment to naturally consider AI-based insights when making decisions. Several participants posited that caregivers are responsible for critically examining what care is needed and appropriate in the context of an individual client and for using all available inputs, including insights generated by AI-DSSs. This might imply that consulting AI-DSSs might become the norm over time as more evidence becomes available about the added value of these systems for the quality and efficiency of care and trust increases. Multiple participants mentioned that AI-DSSs should be adequately integrated into the broader work processes of caregivers to optimally use AI-based insights. A data specialist put this as follows:

I think you should arrange implementations of algorithms in such a way that caregivers cannot work around them. You have to make the process foolproof. For example, as we have done here...We have arranged that every client with a positive outcome on the algorithm must be discussed by the coordinating practitioner and the manager. Then caregivers are still the ones who decide about what happens and the manager is the one who asks questions.

Participant 23

In addition, the participants mentioned multiple factors that are important for the routinization of AI-DSSs. For instance, several participants mentioned that caregivers should have the freedom to deviate from or disregard the outcomes of AI-DSSs, provided that they do so thoughtfully. Accordingly, some participants suggested that it might be essential for caregivers to comprehensively report their decisions and actions in the care process. It was also suggested that care protocols and agreements within care organizations, or the care sector more broadly, regarding the authority and decision-making power of caregivers should be regularly evaluated.

## Discussion

### Principal Findings

This study aimed to gain insights into the perspectives of nurses and other professional stakeholders in LTC on prerequisites for responsible AI-assisted decision-making in nursing practice. By first examining stakeholders’ perspectives on the opportunities and risks of AI-assisted decision-making, the groundwork was established for exploring their perspectives on prerequisites for responsible innovation in this area. As our results demonstrate, the stances of LTC professionals toward the use of increasingly advanced AI-DSSs are not a matter of purely positive or negative expectations but rather a nuanced interplay of positive and negative elements that lead to a weighed perception of prerequisites for responsible AI-assisted decision-making in nursing practice. Our findings provide insights into potential supportive roles of AI-DSSs in nursing practice. For instance, AI-DSSs can elevate the remote and early anticipation of care needs by harnessing data from various sources (eg, care technologies) and swiftly uncovering overlooked issues or emerging trends related to the health, well-being, or behavior of a client. In addition, AI-DSSs are expected to foster adaptive, data-informed decision-making about person-centered care strategies as well as shared decision-making by clients and their formal and informal caregivers. Furthermore, the use of AI-DSSs is expected to alleviate the cognitive load of caregivers and improve their work experience by saving time that would otherwise be spent on repetitive, intricate, and burdensome analytical and monitoring tasks. AI-DSSs are not regarded as potential decision makers in the nursing process but rather as instruments, and by some even as anthropomorphized agents, such as personal coaches or mentors, that could proactively aid caregivers in becoming aware of certain care needs and adaptively responding to these needs. While these perspectives do not necessarily cover the entire spectrum of opportunities of AI-assisted decision-making, they correspond with previous studies on the expectations, opportunities, and applications of AI in LTC (eg, the studies by Mukaetova-Ladinska et al [2], Seibert et al [7], Buchanan et al [8], and Neves et al [47]).

Our findings also provide insight into perceived risks of AI-assisted decision-making in nursing practice. Notwithstanding the positive perspectives regarding the opportunities of using AI-DSSs, the care professionals generally expressed caution about its potential impacts. Despite their limited prior knowledge and expertise regarding the risks of AI, the care professionals shared a diverse array of interrelated concerns about risks associated with AI-assisted decision-making, which mirror findings from previous studies on the ethical implications of using AI-DSSs in health care (eg, the studies by Sutton et al [21], Skuban-Eiseler et al [35], and Schlicht and Räker [36]). For a large part, these concerns revolved around the heavy reliance of caregivers on AI-DSSs, which might, for instance, cause caregivers to overlook crucial nuances that are beyond the grasp of AI-DSSs. AI-DSSs might also perpetuate or exacerbate biases or cause a false sense of security, as certain people and care needs might not be adequately represented in the data and rules that are fed to AI-DSSs. Ultimately, caregivers who heavily rely on AI-DSSs might be led astray toward unsuitable care strategies. These perspectives tie in with how Nyholm [48] sketches the dual effects of AI on human intelligence: the prospect that AI technologies might serve as a form of cognitive enhancement and the cautionary notion that heavy reliance on AI technologies might make people less intelligent. Furthermore, in our study, concerns were expressed related to privacy infringements, conflicts of interest, and the deterioration of the work experience of caregivers owing to increased cognitive load or a reduced sense of professional fulfillment.

Expanding on both the opportunities and risks of AI-assisted decision-making in nursing practice, the care professionals participating in this study were able to articulate factors that might be important for responsibly embedding AI-DSSs into nursing practice. Overall, the reasoning of care professionals about the responsible design, implementation, and use of AI-DSSs in nursing practice centered on seven interrelated categories of prerequisites: (1) regular deliberation on data collection; (2) a balanced proactive nature of AI-DSSs; (3) incremental advancements aligned with trust and experience; (4) customization for all user groups, including clients and caregivers; (5) measures to counteract bias and narrow perspectives; (6) human-centric learning loops; and (7) routinization of using AI-DSSs. These findings extend beyond merely mitigating the risks of AI-DSSs deployment in nursing practices, as they provide insights into the envisioned interactions between people and technology and how these interactions can be responsibly shaped and reshaped as both technology and the needs and values of people evolve.

### Implications for Research and Practice

An overarching lesson to be learned from the identified prerequisites for responsible AI-assisted decision-making in nursing practice is that care professionals perceive that despite the advancing capabilities of AI, AI-DSSs should serve as tools that support shared decision-making by clients and their care networks. Responsible AI-assisted decision-making hinges on mutual reinforcement between users and technology. To maximize the benefits and minimize the negative implications of AI-assisted decision-making, the ways in which AI-DSSs support nursing practice and interact with caregivers and other stakeholders require continuous refinement “in context.” This implies the need to iteratively tailor the design, implementation, and use of increasingly advanced AI-DSSs to the interests, experiences, and roles of individual clients and caregivers in the care process and to the physical care environment.

The prevailing perspective suggests that inundating nurses, care coordinators, and other care professionals with excessive (aggregations of) data could impede, rather than enhance, their decision-making capabilities. This aligns with previous studies that show that too much information [49,50] and insufficient time can lead to information overload [51]. In this line, it is anticipated that the use of AI-DSSs can ease caregivers from data-intensive analytical tasks, proactively direct their attention to issues and trends in data that may need their attention, and possibly even guide them toward certain care strategies (see prerequisite 2). These findings align with previous studies that posit the use of AI as a “technical fix” to mitigate existing risks related to the remote monitoring of older adults, such as the potential cognitive overload of caregivers [32,52]. However, the anticipated utility of proactive AI-DSSs must be carefully balanced against the predominant perspective that the automation of decision-making in nursing practice should be avoided (prerequisite 2); that AI-DSSs might be introduced in practice only through incremental steps that are aligned with users’ evolving trust in, and experience with, using these systems (prerequisite 3); and that vigilance is required to prevent caregivers from becoming overly reliant on AI-DSSs and being led astray toward unsuitable care strategies (see also the studies by Parasuraman and Riley [53] and Goddard et al [54]). In this regard, our findings highlight the importance of and ways to actively counteracting bias and narrow perspectives during both the design and use of AI-DSSs (prerequisite 5; see also the studies by de Hond et al [55], Fosch-Villaronga et al [56], and Rubeis [57]). These findings complement previous studies showing that AI tools can contribute to the over-problematization and overdiagnosis of health issues [58] and perpetuate racial, gender, and age-related biases [24,47,59,60]. Moreover, in close connection to this, our findings emphasize the importance of establishing human-centric learning loops through which caregivers can actively contribute to the meaningful and responsible design, implementation, and use of AI-DSSs (prerequisite 6) [57,61]. These findings resonate with Hindocha and Cosmin Badea [34], who suggested that care professionals can act as moral exemplars for the virtuous machine and will, therefore, be integral to the responsible design, deployment, and use of AI in health care. Moreover, caregivers play an important role in collecting data that might eventually be used by AI tools [61]. Overall, these findings underscore the notion that responsible AI-assisted decision-making requires an approach that extends beyond merely the design and technical aspects of AI-DSSs. The development and use of AI-DSSs should be supported by caregivers capable of adeptly interacting with these technologies (see also the study by Sand et al [62]). The enhancement of capabilities calls for effective educational strategies to prepare caregivers for this evolving technological landscape [63]. However, as our findings suggest, caregivers may not contribute equally to responsible innovation in this area. Although all caregivers are obliged to justify their own decisions and actions [64], some may need practical assistance in the optimal and responsible use of AI-DSSs. Meanwhile, other caregivers can take on active intermediary roles between care and technology [61] by providing practical assistance to fellow caregivers and supporting designers in shaping and iteratively improving AI-DSSs.

Although our findings suggest that the overall potential of AI and AI-DSSs grows with the availability of pertinent data, they also show reservations against the unrestrained collection and use of data by AI-DSSs. The predominant perspective of care professionals was that specific data and associated AI-based insights should be generated only in accordance with established goals agreed upon by key stakeholders, including clients (prerequisite 1). The collection and use of specific data should be proactively and continuously balanced against potential harms, such as privacy infringement, cognitive overload, and the over-problematization of old age (see also the studies by Wang et al [65], Blasimme and Vayena [66], and Palmer and Schwan [67]). Although our findings emphasize the importance of generating only relevant data as input for AI-DSSs, they also suggest that once it has been decided to generate certain data and have them processed by AI-DSSs, it should be routine practice to use the resulting insights (prerequisite 7). In this context, Heyen and Salloch [22] stressed that the more routinized the use of AI-DSSs becomes in practice, the more critically caregivers need to focus on soft factors in individual client cases that cannot be comprehensively considered by AI-DSSs, such as the personality, life situation, or cultural background of a client (see also prerequisite 5). Similarly, a notable skepticism was present among the care professionals participating in this study regarding the future capacity of AI to comprehensively anticipate the care needs of people. After all, it may be difficult or even impossible to fully capture in data and decision rules for AI what contributes to good care and quality of life for an individual person [35,36,68]. Hence, in the context of AI-assisted decision-making, it may become increasingly important to engage in shared decision-making to get to know clients and respond optimally to their personal needs, goals, interests, preferences, and values [22,69]. Simultaneously, the shared decision-making model is subject to pressure, for instance, owing to the potential opacity of algorithms, leading to an insufficient understanding of the rationale behind AI-based insights into care needs and possible interventions [64]. Moreover, shared AI-assisted decision-making may be particularly challenging in the care of older adults, particularly those with cognitive impairment. This may hinder the ability of older adults to express their feelings and wishes and amplify the risk that nurses and other formal and informal caregivers consciously or unconsciously enforce what they think is right [35,36,70,71]. A fruitful direction for future studies could be to explore the effective integration of AI-DSSs into shared decision-making processes with older adults and their formal and informal caregivers.

### Responsible Innovation: A Balancing Act

As our findings and the implications drawn earlier indicate, initial opportunities for AI-assisted decision-making in nursing practice could turn into drawbacks, contingent upon the specific shaping of both the design and deployment of AI-DSSs. The interrelatedness of the identified prerequisites for responsible AI-assisted decision-making suggests that addressing one factor alone may not be sufficient because of its tight link with others. Moreover, addressing risks such as privacy infringement, for instance, by limiting data collection, affects the possibilities of remote care and prevention supported by AI. Hence, we call for technology developers; caregivers using AI-DSSs; and other stakeholders, including older adults, to engage in ongoing public discourse (see also the study by Buhmann and Fieseler [72]) and work together to *cohesively* address different factors important to the responsible embedding of AI-DSSs in practice. In doing so, we recommend viewing the responsible use of AI-DSSs as a *balancing* *act* (see also the study by Wehrens et al [52]). Potential or proven positive and negative impacts could be carefully weighed against each other, or stated differently, trade-offs could be made among the effects of using AI-DSSs on values such as quality of life, autonomy, privacy, transparency, and fairness (see also the study by Sanderson et al [73]). Further research could explore at what level and by which means such trade-offs can be made effectively.

While trade-offs need to be made in context, in the care of individual clients, there are also trade-offs to be made at a higher level between the interests of individual people and broader public interests. Our findings suggest that responsible AI-assisted decision-making requires customization, for instance, regarding specific care technologies to be used and data to be collected [74], the processing of these data by AI, who gets access to the data and AI-based insights, the explanation of AI-based insights to users [75], and the extent to which AI-DSSs proactively advise caregivers about care needs and strategies (see prerequisite 4). In other words, there might be a desire to comprehensively address context-specific needs and preferences regarding privacy protection, transparency about the outcomes of AI-DSSs, and the protection of caregivers from potential overreliance on AI-DSSs and the erosion of professional autonomy (eg, the studies by Egelman and Peer [76] and Wilkinson et al [77]). One might suggest that the responsible deployment and use of AI-DSSs in practice requires customization at the level of individual clients and caregivers. Simultaneously, full customization might be at odds with the need to offer somewhat standardized solutions, universalize applicability, and foster scalability [78-80]. Future studies could explore how trade-offs could be made between the seemingly contrasting needs for contextualization and customization and for the decontextualization and standardization of AI-DSSs. In addition, it would be valuable to examine the implications of such trade-offs for the development of AI-DSSs and their deployment in practice.

Several studies have been conducted on the (potential) supportive roles of AI-based technologies in nursing practice [5-8] and the high-level requirements for responsible AI innovation [28-30]. This study builds upon previous studies in both research fields by examining the perspectives of various experienced nurses and other LTC professionals on the opportunities and risks of AI-assisted decision-making in nursing practice, thereby laying the groundwork for exploring associated prerequisites for responsible innovation in this area. This is particularly relevant because nurses and other caregivers do not always have a say in the design of AI tools, while they play a pivotal role in their implementation and use [5,8,57,61]. Along this line, we recommend that future studies continue to engage with the perspectives of caregivers and other stakeholders on striking a balance between the opportunities and risks of AI-assisted decision-making. This could contribute to a more comprehensive analysis and deeper understanding of ways to ensure the responsible embedding of AI-DSSs and other AI-based technologies in specific contexts. Another avenue worth exploring in future studies involves the demonstration of effective methodologies and metrics for an in-depth evaluation of the positive and negative impacts of AI-DSSs on the dynamics of nursing practices and the tensions between these impacts. Research endeavors of this nature could offer initial steps for diverse stakeholders in working together on the responsible embedding of specific AI-DSSs in practice.

### Study Limitations

No study comes without limitations, and the main limitations of this study are related to the participants involved. For instance, by focusing *only* on the perspectives of LTC care professionals, this study does not consider the perspectives of *other* key stakeholders in AI-assisted decision-making in LTC, such as older adults and informal caregivers. Moreover, within LTC, an increasing number of caregiving responsibilities may transition to informal care networks. This highlights the need for future studies to include both formal and informal caregivers and care recipients to gain a comprehensive understanding of the prerequisites for responsible AI-assisted decision-making in nursing practice [36]. In addition, our findings may guide responsible innovation in AI-DSSs outside the context of Dutch LTC, but caution should be exercised in generalizing our findings, given the diversity of health care systems across countries. The results obtained from this study can be further examined in future studies using a quantitative approach or a larger and more diverse sample of LTC stakeholders from different geographic and cultural backgrounds, thereby evaluating and enhancing their robustness. Furthermore, despite the diverse group of care professionals participating in this study, biases may exist owing to varying experiences with digital innovation, potentially skewing views toward the desirability and implications of AI-assisted decision-making. In addition, the targeted (principle-based) interview questions may have influenced the responses of participants by guiding specific conceptualizations of risks. While this guidance may have positively contributed to gaining in-depth insights into prerequisites for responsible innovation, it may also have caused omissions of crucial factors, such as the impact of AI on the environment, digital inequality, and the caregiver-client relationship, which should also be considered in contexts of AI-assisted decision-making. Finally, to enhance the comprehension of the prerequisites for responsible AI-assisted decision-making, future studies might also consider and enlighten sociotechnical biases and potentially skewed perceptions of care professionals and other stakeholders about the opportunities and risks presented by AI-DSSs (eg, see the study by Neves et al [47]).

### Conclusions

This study provides insights into prerequisites for responsible AI-assisted decision-making in nursing practice from the perspectives of nurses and professional stakeholders with whom they closely collaborate. While care professionals see broad opportunities in the use of AI-DSSs to improve the quality of care and workload and experience of caregivers, positive perspectives on AI-assisted decision-making are generally accompanied by a wide array of concerns about risks. Our findings indicate that opportunities for AI-assisted decision-making in nursing practice could turn into drawbacks depending on the specific shaping of the design and deployment of AI-DSSs. To optimally balance opportunities and risks of AI-assisted decision-making, seven interrelated categories of prerequisites were identified for responsible AI-assisted decision-making in nursing practice: (1) regular deliberation on data collection; (2) a balanced proactive nature of AI-DSSs; (3) incremental advancements aligned with trust and experience; (4) customization for all user groups, including clients and caregivers; (5) measures to counteract bias and narrow perspectives; (6) human-centric learning loops; and (7) routinization of using AI-DSSs. These prerequisites emphasize that regardless of their advancing capabilities, AI-DSSs should be used as tools to support shared decision-making by clients and their care network, and the ways in which AI-DSSs support the nursing process need continuous contextual refinement. Although this study focuses on the use of AI-DSSs in LTC, the findings may also be relevant to different sectors, contexts, and AI-based technologies. Finally, this study demonstrates the relevance of engaging care professionals in exploring the opportunities and risks of AI, as well as factors important to the responsible embedding of AI-based technologies into practice. These actors not only play a pivotal role in the future use of AI-based technologies in care practice but can also actively contribute to the articulation of strategies that ensure meaningful, responsible, and sustainable embedding of technologies in practice.

## Appendix

Multimedia Appendix 1 Interview protocol (translated to English).

## Abbreviations

AI: artificial intelligence
AI-DSS: artificial intelligence–based decision support system
HAAL: Healthy Ageing Eco-system for People With Dementia
LTC: long-term care
WHO: World Health Organization
