# Supplementary material for: AI-Assisted Decision-Making in Long-Term Care: Qualitative Study on Prerequisites for Responsible Innovation
Source: JMIR Nurs. 2024 Jul 25;7:e55962. doi: 10.2196/55962 (PMC11310645; doi:10.2196/55962)
Supplement: Multimedia Appendix 1 [file nursing_v7i1e55962_app1.docx]

Interview protocol

**Explanation about the colouring of questions:**

- The text in black is an explanation that should be read literally to the respondent.
- **The green-printed (numbered) questions should all be asked, in chronological order.**
- The blue-printed questions (a, b, c, etc) are optional additions to the numbered questions. These optional questions can be asked at the interviewer's discretion, for instance when respondents have a lot to tell about the respective theme, or as 'probing questions' to help the respondent get started with an answer when they initially find it difficult to answer.
- Please be vigilant during the interview that you as an interviewer are not too steering, and that you allow the respondent to share as much as possible about what they believe is important.
- During the interview, PowerPoint slides can be used to show the images.

Responsible Innovation in HAAL

Part 1

Introduction

Thank you for joining the interview. This is very valuable for the HAAL project because we like to match the technology as closely as possible with care practices and the people who will eventually use it or affected by it.

Can we record the interview just for ourselves to process the data?

The interview will last between 1 and 1.5 hours. There are quite many questions we want to ask you. Therefore, we will to run through some parts of the interview pretty quickly. The first part of the interview is meant to get to know you, and to introduce you to the project. The second part delves deeper into the ethical and social impact of technology.

*<turn on recording & transcript>*

Let us introduce ourselves first. And then there are some questions about you and your work situation.

*<researchers introduce themselves>*

1. ***What is your job title? And can you shortly explain what this job entails?***
2. ***What is your age?***
3. ***How long have you been working in care for people with dementia?***
4. ***On average, how often do you work with people with dementia? Daily/weekly/monthly/yearly/barely***
5. ***Can you briefly tell us more about the role you play in their care?***
6. ***In which phase of dementia are the clients you mainly care for?***

Overview of technologies

In short, within the HAAL project we are investigating how a bundle of care and welfare technologies can be used for people living at home with dementia. In the image below you can see the different technologies that are part of the HAAL project. I will briefly explain them. *<show flowchart>*

**
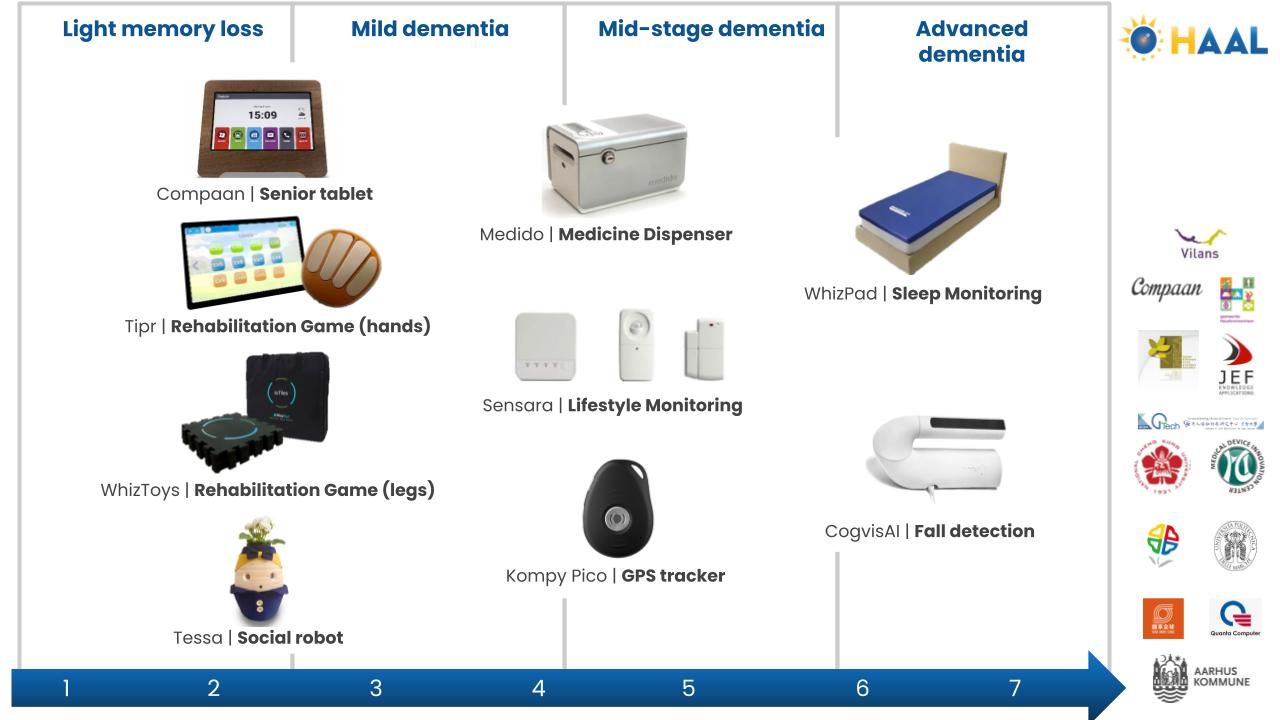
**

1. de Compaan, a seniors tablet with which you can, for example, view social online contact of the client.
2. Medido, a medicine dispenser, with which you can see whether the client is taking his medication.
3. Whizpad, a smart mattress, with which you can see, among other things, how well the client sleeps, time in bed, how often he leaves his bed, and whether there is a risk of bedsores.
4. Tipr, a hand-drag rehabilitation game that allows us to see cognitive decline.
5. Sensara, sensors for the home, with which you can see in which room the client is located.
6. Whiztoys, also a game that allows us to see cognitive decline.
7. Cogvis AI, a fall detector, which measures, among other things, the danger of falling.
8. Tessa, a social robot who provides daily structure, can ask questions.
9. GPS tracker, which knows where the client is and can measure activity.

**Familiarity with the technologies**

1. ***Do you know one or more of these technologies, or similar products? Do you or your clients already use them themselves?***
   1. *Check: Do you have any questions about any of the technologies or the data generated by the technologies?*

Part 2

HAAL dashboard

The different technologies all generate data that directly or indirectly tell something about the health, well-being or safety of community-dwelling people with dementia. Such data can, for example, help caregivers assess and determine the care and support needs of their clients. Within the HAAL project we are developing a dashboard in which we make the data from the various technologies available in one place. The dashboard is in fact a tool for linking the data back to caregivers (and any other stakeholders) in a manageable way so that they can do something with it. To investigate how we can properly design this dashboard, and how it can be applied in practice, we conduct these interviews.

1. ***Now that you have heard this information, what information (data) would you find interesting to see in the dashboard?***

**(Note: We are not interested specifically in the function of the technological tools, but rather in the data they generate.)**

- 1. *Why do you consider this information to be relevant?*
  2. *How would this information affect your work? For example, which interventions would you deploy based on this data?*
  3. *Do you have any other information that you find relevant?*

1. ***For whom else then yourselves do you think this dashboard could be relevant? Are there any types of colleagues in your organization, or people outside your organization such as the client himself, for whom such a dashboard can be useful? Why?***

To make it more tangible and understandable what the HAAL dashboard could look like, we have made up two scenarios. Both scenarios are fictional and intended to inspire you about what the dashboard might look like.

Scenario 1

Scenario 1 is about a low-complex, descriptive dashboard. Below you see an example that illustrates the scenario. *[show illustration in powerpoint]*


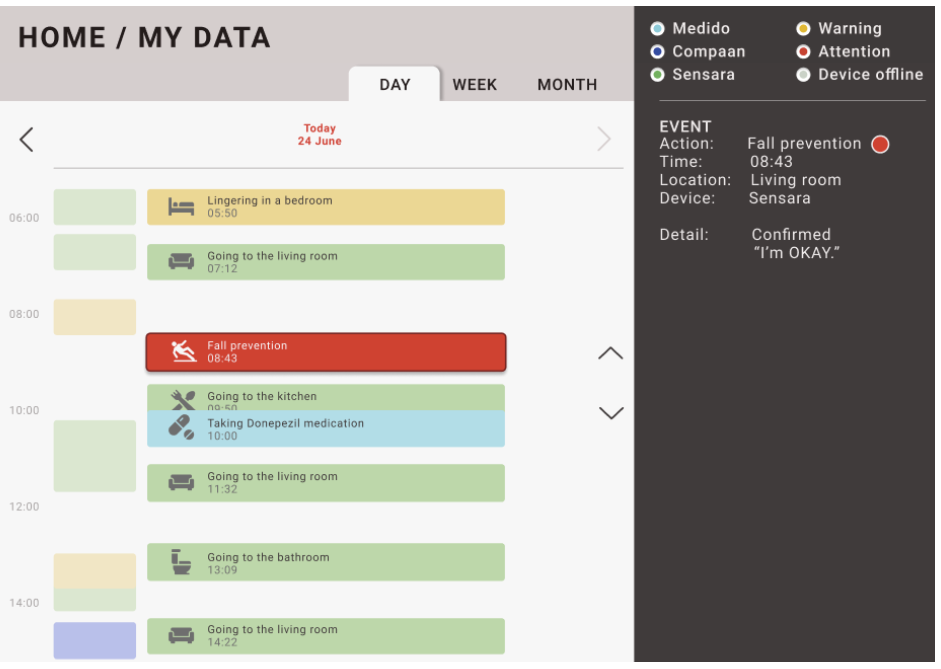


The dashboard provides a picture of the current situation in the client's home based on data about e.g. exercise, nutrition, medication intake and social contact. The dashboard is mainly intended to make various data available to healthcare providers in one place, so that they do not have to look in separate overviews and apps. Important about this scenario is that no major interpretation of the data has yet been made in the dashboard, except that colours are used to indicate, among other things, whether there is potentially a risky situation (red), a situation that requires attention (orange) or if there is no action appears to be required (green). of the descriptive dashboard.

1. ***To what extent do you think such a descriptive dashboard could be of added value in home care for older people with dementia?***
   1. *What do you think of the way in which data and information is fed back to the user, i.e. of the interpretation that the dashboard makes on the data?*

Scenario 2

Here you see an illustration for scenario 2. *[show illustration in powerpoint]*

**
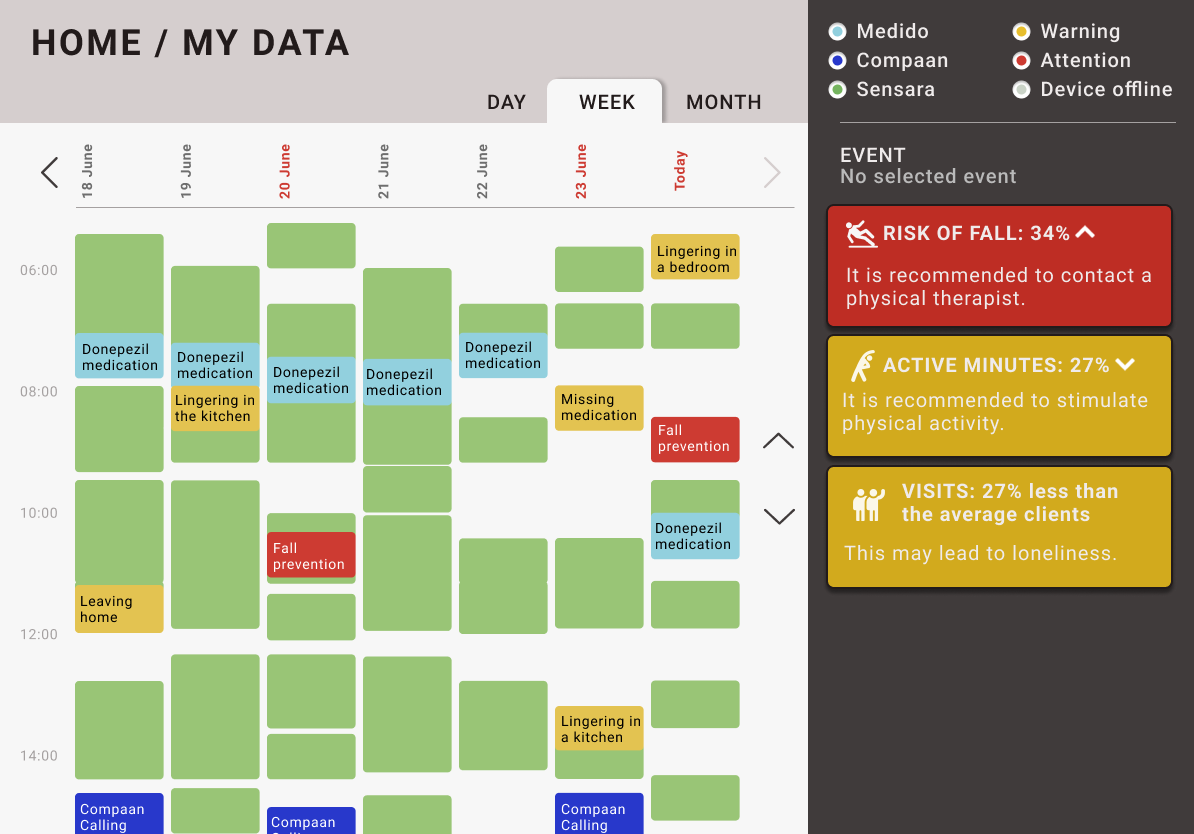
**

Scenario 2 is an advanced dashboard that automatically translates the data into insights and recommendations for healthcare providers. In this collaborative dashboard, for example, based on data, warnings or risk scores are automatically displayed about possible emergency situations such as a fall. And the dashboard makes recommendations on certain possible follow-up actions, for example to stimulate a client's physical activity if the data shows that they have been relatively inactive in the past few days.

1. ***To what extent do you think such a proactive and collaborative dashboard could be of added value in the home care for older people with dementia?***
   1. *What do you think of the way in which data and information is fed back to the user, i.e. of the interpretation that the dashboard makes on the data?*

Role of AI

Regardless of whether the actual development of the HAAL dashboard will move more towards scenario 1 or 2, AI will play a central role. The term AI stands for artificial intelligence and refers to the attempt to mimic parts of human intelligence in machines. With AI you can think about computers that make predictions, recommendations or decisions based on data and decision rules that are programmed into the systems. An algorithm tells the computer which specific steps must be performed in a specific order to achieve a certain end result.

1. ***Do you understand what AI is? And if so, what examples of AI come to mind from everyday life? These don't need to be examples in healthcare.***

*<Depending on their answers, you can give examples, such as:*

- *voice assistant Siri from Apple (including speech-to-text)*
- *Google search engine (including both the search results and the word suggestions you get when you type a few letters in Google)*
- *navigation on Google Maps*
- *personalized timeline and suggestions on Facebook, Spotify, Netflix*
- *spam filters in your mailbox*
- *drones that monitor crop growth or count livestock in agriculture*
- *image interpreting cameras that can determine if someone has fallen>*

1. ***What role do you hope AI will have in your work?***

Comparison scenarios

The figure below shows a simple representation of how the two scenarios we just discussed differ from each other in terms of complexity and the extent to which AI plays a role. *[show figure in powerpoint]*


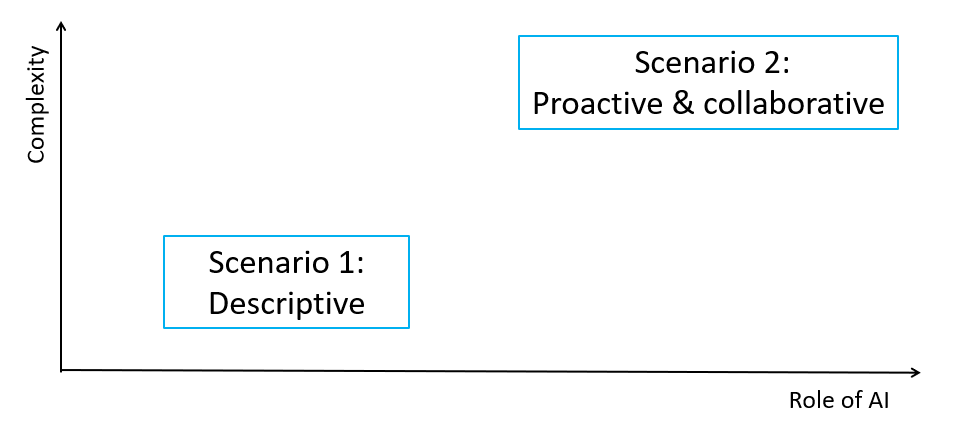


In scenario 1, AI plays a minor role. Algorithms determine here, for example, with which colour certain information is marked, but apart from that, the interpretation by the computer and the complexity of the analyses is low. Therefore, it may take a little more thinking to extract meaning from the data.

In scenario 2, AI plays a major role. Based on data, the dashboard automatically makes predictions and recommendations about follow-up actions. The collaborative dashboard has a higher complexity and based on predictions and recommendations, this dashboard takes some of the cognitive workload over from the caregiver.

1. ***If you had to choose between scenario 1 and 2, which one would you prefer?***
2. *Why?*
3. *<repeat the reason they give and ask again why this is important>*

Part 3

Risks and downsides

During the HAAL project, we investigate how we can use AI in a meaningful and responsible way as decision support for caregivers and other stakeholders. We also pay attention to the ethical aspects of AI, because the use of AI in healthcare offers many opportunities, but also entails certain risks.

1. ***Can you think of any risks or disadvantages that come with using a dashboard like in scenario 1 or 2?***
2. *For whom are these risks / disadvantages?*
3. *Why? (for example, privacy can be a risk for several reasons, so it's interesting to ask what's behind it)*
4. ***Do you see any differences between scenario 1 and 2 in terms of risks or disadvantages? If so, which one?***
5. ***What are your first thoughts about how to deal with the discussed risks or disadvantages? Do you have any thoughts about how the developers of the dashboard, or users themselves, can deal with this?***

Targeted questions about responsible innovation

We first asked you openly about the risks and points of attention that you see for the responsible use of the HAAL dashboard. We will now also ask you about a few themes that are important to take into account when deploying AI. For each theme, we first briefly tell you something about the theme, and then ask you what risks you see in this area. Please tell as much as possible about what comes to mind when we discuss the themes. We ask you about ‘risks’ related to each theme. If something does not really feel like a big risk to you, but rather like a ‘point of attention’ – i.e. something to be aware of and take into account – then it is just as relevant to share with us.

1 Inclusion and equality

One theme to consider when deploying AI is inclusion and equality. AI systems must be designed and deployed in such a way that they can support the broadest possible target group (i.e. people of various ages, backgrounds and personal characteristics) and that the datasets they learn from, for example, are not biased and therefore disadvantage certain individuals or groups of people. If, for example, the HAAL dashboard learns from data of mainly older clients, there is a risk that the system makes unreliable predictions and recommendations about younger clients (e.g. people diagnosed with young onset dementia).

1. ***Can you think of any other factors to take into account to make the HAAL dashboard representative and fair?***
2. ***Do you see any risks with respect to inclusion and equality when developing and using the HAAL dashboard?***
   1. *Are these risks different for scenario 1 than for scenario 2? If so, how?*
3. *What are your thoughts about how to deal with the discussed risks?*

2 Human autonomy

Another important theme in AI is human autonomy. The use of AI systems can lead to situations in which certain decision-making, or steps in decision-making, are partly or even completely transferred to technology.

1. ***Do you think that the use of the HAAL dashboard can have an impact on the autonomy of users? Do you see any risks in this area?***

<If respondents doubt or answer ‘no’, you can explain: even if technology only makes recommendations, it consciously or unconsciously quickly influences the decisions you make as a person. Moreover, AI is often something that mainly plays a role in the background. As a user, you often do not see (or only to a limited extent) how certain input is converted into results by algorithms. Think for example of the search engine of Google: as a user you do not see how the system determines which results you get for a certain search.>

- 1. *What do you think of this in the context of the HAAL dashboard? Could it affect your autonomy as a caregiver?*

1. *And do you also see a possible influence on the autonomy of the client?*
2. *Do you see any differences between scenario 1 and 2 in terms of risks regarding people’s autonomy? If so, which ones?*
3. *What are your thoughts about how to deal with the discussed risks or disadvantages?*

3 Transparency and explainability

We are also interested in your view on the transparency and explainability of the outcomes of AI. Many AI systems are complex, which means that it is not always entirely clear how their outcomes come about. The performance of AI systems often improves as they can learn from larger amounts of data - because more data often means more evidence on which outcomes are based. But this is often at the expense of transparency and explainability of AI – it is then not clear, or only to a limited extent, how AI processes certain inputs into outcomes.

1. ***What would be more important to you?; that the dashboard shows you the most accurate recommendations OR that you can fully understand the recommendations provided by the dashboard and interpret how the system came to these insights? Can you elaborate why?***
2. ***Do you see any risks with respect to transparency and explainability in the HAAL dashboard?***
   1. *Are these risks different for scenario 1 than for scenario 2? If so, how?*
   2. *What are your thoughts about how to deal with the discussed risks or disadvantages?*

4 Responsibility and liability

While AI systems perform specific tasks, for example by making predictions or recommendations, the question is who can take responsibility for the consequences of those outcomes of AI.

Of course, it is possible that technology like the HAAL dashboard sometimes points users in the wrong direction. For instance, imagine a scenario where based on the currently provided information and recommendations from the HAAL dashboard, a caregiver concludes that no additional measures are needed to reduce the risk of falling for this client. The next week this turns out to be an adverse decision, because the client fell anyway.

1. ***Who do you think should be liable if a decision made based on the dashboard turns out to be an adverse decision about the care for a client? In other words, who do you think should is responsible if something goes wrong when using the HAAL dashboard?***
2. ***Do you see any risks with respect to responsibility and liability?***
   1. *Are these risks different for scenario 1 than for scenario 2? If so, how?*
   2. *What are your thoughts about how to deal with the discussed risks?*

5 Well-being and safety

AI systems should of course not have negative consequences for people. They must be safe, accurate and effective before being deployed.

1. ***Do you see any risks in this area?***
   1. *Are these risks different for scenario 1 than for scenario 2? If so, how?*
   2. *What are your thoughts about how to deal with the discussed risks or disadvantages?*

6 Align with, and adapt to circumstances

Furthermore, AI systems must of course match the context of and circumstances in practice, even if these change over time. In fact, they should be developed and introduced only if they can be adequately and fully integrated in the care process.

1. ***Do you have any ideas about what in this sense would be needed to ensure that AI systems match the needs and context of care practice?***
2. *Do you see any risks?*
   1. *Are these risks different for scenario 1 than for scenario 2? If so, how?*
3. *And what are your thoughts about how to deal with the discussed risks?*

Closing the interview

We are at the end of this interview.

1. ***Do you have any other suggestions or things you would like to discuss about the social and ethical impact of the HAAL dashboard?***

We use the results of the interviews to reflect, together with the other organizations we collaborate with in this project, on what is important for the meaningful and ethical application of the HAAL dashboard. We try to incorporate the insights from users directly into the development process. We will also write publications based on the results, so that other innovators and users of AI applications in healthcare can also learn from our lessons about responsible innovation.

**Optional**

1. ***Do you know of any other healthcare professionals that we could interview? These can be different types of professionals for which such a dashboard could be relevant, for example district nurses, geriatric specialists, but also data experts, for instance.***
2. ***May we eventually approach you more often in later research to explore this topic in more depth?***

<Thank you and goodbye>
